# Expression patterns of estrogen and androgen receptors in NSCLC patients according to the PD-L1 profile

**DOI:** 10.3389/fimmu.2025.1602579

**Published:** 2025-06-19

**Authors:** Vianey Rodriguez-Lara, Gala Cortés-Ramírez, Itzel Amayrani Angeles-Torres, Jeronimo Rodriguez-Cid, Sally María Luisa Pedraza-Reyes, Maria Rosa Avila-Costa, José Luis Ordoñez-Librado, Marco Cerbón

**Affiliations:** ^1^ Department of Cell and Tissue Biology, Faculty of Medicine, UNAM, Mexico City, Mexico; ^2^ Department of Evolutionary Biology, Faculty of Science, UNAM, Mexico City, Mexico; ^3^ Neuromorphology Laboratory, FES Iztacala, UNAM, Mexico City, Mexico; ^4^ Department of Thoracic Oncology, Instituto Nacional de Enfermedades Respiratorias, Ismael Cosio Villegas, Mexico City, Mexico; ^5^ Department of Biology, Faculty of Chemistry, UNAM, Mexico City, Mexico

**Keywords:** estrogen receptor, androgen receptor, NSCLC, PD-L1, immunotherapy

## Abstract

**Background:**

Lung cancer is the leading cause of cancer-related death worldwide, with non-small cell lung cancer (NSCLC) being the most common type. Immunotherapy targeting programmed death ligand-1 (PD-L1) blockade has significantly improved survival, but differences in responses by sex have been reported, suggesting a possible role of sex hormones. Estrogens and androgens, through their receptors support lung carcinogenesis, but their role in immune evasion via the PD-1/PD-L1 pathway remains poorly understood.

**Materials and Methods:**

We analyzed by immunohistochemistry the expression patterns of estrogen receptors (ERα and ERβ) and androgen receptor (AR) in 95 PD-L1-positive (PD-L1+) and 72 PD-L1 negative (PD-L1-) NSCLC patients by sex and hormonal status. We also investigated associations between hormonal receptors, PD-L1 profile, PD-L1 tumor proportion score (TPS), and clinical features (cancer stage according to the TNM stage of cancer, smoking history, wood smoke exposure, and asbestos exposure).

**Results:**

ERβ was the predominant form of estrogen receptor in PD-L1- patients, while ERα expression was significantly higher in PD-L1+ patients and strongly associated with the PD-L1+ profile, regardless of sex or hormonal status. AR expression was low across all groups and showed no association with PD-L1. Among PD-L1+ patients, ERα expression levels were highest in premenopausal women, followed by men and postmenopausal women. ERα levels in the PD-L1+ group, were not associated with PD-L1 TPS or with clinical features.

**Conclusion:**

The estrogen pathway, particularly via ERα, plays a key role in PD-L1 expression and may contribute to tumor immune evasion. Antiestrogen therapy could represent a promising strategy to enhance immunotherapy efficacy in patients expressing ERs.

## 1 Introduction

Lung cancer (LC) continues to be the leading cause of cancer-related death in men and is the second leading cause in women worldwide, representing a relevant health problem globally. The predominant type is non-small cell lung cancer (NSCLC), accounting for 85% of diagnosed cases (1, 2). NSCLC exhibits a different behavior by sex; women as well as young people and non-smokers are more likely to be diagnosed with lung adenocarcinoma, while men and smokers frequently develop squamous subtype. Although tobacco smoking is one of the main risk factors related to lung cancer, a higher proportion of female never-smokers develop lung cancer (3, 4) and are diagnosed at younger ages compared to men (5). In the United States, the incidence and mortality rates in male patients are decreasing; however, this trend is slower in women. Indeed, lung cancer incidence in young women (<65 years) exceeded that observed in men in 2021 (6). Among female smokers, a higher risk of developing lung cancer at the same level of smoking was reported compared to men. Women are more susceptible to tobacco carcinogens exhibiting higher DNA adducts and reduced DNA repair capacity. Differences in the detoxification system by p450 proteins have also been observed; women overexpressed cytochrome CYP1A1 resulting in the bioactivation of tobacco carcinogens (7–9). In addition, the prevalence of mutations in EGFR and KRAS, is higher in females than in male patients (10).

Differences in survival and treatment responses have also been reported. Female NSCLC patients exhibit higher overall survival (OS) and a lower risk of death compared to men (11, 12). Moreover, Sachs et al. (13), reported a lower survival advantage in women under 60 compared to older women and men. Furthermore, postmenopausal women showed longer OS compared to premenopausal patients (31.1 *vs* 19.4 months) (14), supporting the significant role of sex and hormonal status in biological features and clinical behavior of lung cancer.

Given these observed differences in clinical outcomes, understanding the role of sex-related factors in the response to treatment becomes crucial, especially considering recent advances in LC therapy. Immunotherapy based on controlling the programmed death protein 1 (PD-1) and its ligand PD-L1, is currently the gold standard for LC treatment (15). PD-1 is a transmembrane protein expressed on numerous immune cells, and after binding to its ligand PD-L1/2, it controls T cell proliferation and its activity. In tumors, cancer cells express PD-L1 to block T cell effector activity and evade the immune system (16). PD-L1 inhibitors have significantly improved the survival of patients, regardless of the PD-L1 tumor proportion score (PD-L1 TPS) (15, 17). However, several studies have shown differences in responses by sex, with greater benefit in male compared to female patients, especially when immunotherapy was applied as monotherapy (15, 18–20). Recently was reported worse progression-free survival (PFS) and higher toxicity in women treated with immune checkpoint inhibitors (ICIs) (including anti-PD-L1) compared to men, mainly in patients with lower body mass index and lower tobacco exposure, but no differences in OS were observed by sex (21). In contrast, some authors found no differences in responses to ICIs by sex (22, 23), although differences in predictors to response (24) and toxicity (23) was reported. Sex bias in outcomes and adverse effects of ICIs is a field of growing research, and future studies are needed to clarify these observations and to elucidate the mechanisms that support sex differences in responses to immunotherapy. An emerging hypothesis suggests a relationship between sex hormones and the PD-1/PD-L1 pathway.

The role of estrogen in lung cancer has been widely investigated. Estrogen (E2) through its nuclear receptor alpha (ERα) and beta (ERβ), activates several pathways involved in lung carcinogenesis, including cell proliferation, tumor growth, antiapoptotic signals, angiogenesis, migration, and metastasis (25–27). Furthermore, estrogen signaling has been associated with proteogenomic alterations and DNA hypomethylation in never-smoker lung adenocarcinoma patients (without EGFR and ALK alterations) (28). Moreover, estrogen regulates the immune response to tumors by stimulating the expression of proteins that activate immune system cells (29). Recently, estrogen has been associated with PD-L1 through its nuclear receptors, but this functional association has not been completely elucidated and remains scarcely explored in patients.

Conversely, the effects of testosterone and androgen receptor (AR) in lung cancer are still unclear. Some studies suggest that AR overexpression is associated with a better prognosis and improved survival. However, other studies have reported increased proliferation, decreasing apoptosis, migration and metastases upon AR downregulation. Additionally, higher survival was observed in NSCLC patients with androgen deprivation therapy, while worse outcomes were reported in patients with AR tumor expression. Androgens, through AR, promote cell proliferation, upregulation of genes related to oxygen transport and DNA repair, and a crosslink between AR and the EGFR and KRAS pathways has been reported (15, 30). Although male lung cancer patients benefit more from immunotherapy based on PD-L1 blockade, the possible association between PD-L1 and the androgen pathway has not yet been explored.

Lung cancer exhibits sex disparities and sex hormones have an essential role in cancer behavior as well as in the response to treatment. Differences by sex in the response to immunotherapy based on blocking PD-L1 have been reported and the effect of hormonal pathways on PD-L1 expression has recently been explored. To date, no studies have described the expression of sex hormonal receptors, both androgenic and estrogenic in PD-L1-positive lung cancer patients. This study identifies ERα, ERβ and AR expression patterns in patients with lung cancer and PD-L1 negative and positive profiles and analyzing whether the association between these hormonal receptors and PD-L1 expression exists. Exploring the relationship between hormonal pathways and PD-L1 could reveal new immune evasion mechanisms mediated by sex hormones and explain sex differences in immunotherapy responses suggesting new strategies to improve treatments.

## 2 Materials and methods

### 2.1 Patients and tissue sample selection

Biopsy tissues from patients with NSCLC were retrospectively selected from the Oncology and Pathological Anatomy Departments at the National Institute of Respiratory Disease (INER) Ismael Cosio Villegas (Mexico City, Mexico). The included patients were diagnosed between 2018 and 2024 and had no initiated treatment at the time of biopsy. Clinical data including sex, age at diagnosis, menopausal status, cancer stage according to the TNM stage of cancer, smoking history, wood smoke exposure and asbestos exposure, were collected from clinical records. Patients without a complete medical history or insufficient tissue in paraffin blocks were excluded. In addition, women who had undergone oophorectomy or received hormone replacement therapy were excluded from this study.

Menopause was defined according to the international menopause guideline as the permanent and natural cessation of menses for 12 or more months. According to standardized guidelines for smoking measurement, a patient was classified as a smoker if they had smoked 100 or more cigarettes in their lifetime and currently smokes, and as a non-smoker if the person had either never smoked at all, had never been a daily smoker, or had smoked less than 100 cigarettes in their lifetime.

This study was conducted following the Declaration of Helsinki and was approved by the Research and Ethics Board of INER and the Faculty of Medicine, UNAM, Mexico. Clinicopathological data from the patients are summarized in **Tables 1** and **2**.

**Table 1:** Clinical features of PD-L1- patients.

PD-L1 - PATIENTS (n=72)			
Sex	Women		Men
Hormonal status	Premenopausal n=21	Postmenopausal n=29	n=22
Age (years) Range	34-50	55-82	29-83
Smoking habit Smoker Non-smoker Pasive smoker	5/ 23.8% 14/ 66.6 % 2/ 9.5%	5/ 17.2 % 19/ 65.6 % 5/17.2%	13/59% 6/27.3% 3/13.7%
Wood smoke exposure	3/14.3%	7/24.13%	4/18%
Asbestos exposure	2/9.5%	2/6.8%	2/9%
TNM I II III IV	0% 1/4.8% 2/ 9.5% 18/85.7%	5/17.3% 1/3.4% 23/79.3% 0%	1/4.5% 0% 4/18.8% 17/77.3%

**Table 2:** Clinical features of PD-L1+ patients.

PD-L1 + PATIENTS (n=95)			
Sex	Women		Men
Hormonal status	Premenopausal n=17	Postmenopausal n=36	n=42
Age (years) Range	35-50	55-80	35-80
Smoking habit Smoker Non-smoker Pasive smoker	3/17.64% 13/76.48% 1/ 5.88%	11/30.5% 23/ 64% 2/ 5.5%	25/59.52% 17/40.47% 0%
Wood smoke exposure	5/ 29.41%	17/ 47.22%	7/16.6%
Asbestos exposure	1/ 5.8%	3/ 8.3%	1/2.3%
TNM I II III IV	0% 0% 2/ 11.77% 15/88.23%	3/8.33% 3/8.33% 2/5.55% 28/77.77%	0% 1/ 2.3% 5/11.9% 83.33%

### 2.2 PD-L1 detection and study groups

PD-L1 expression in tumors was identified by automated and standardized immunohistochemistry using the VENTANA- PD-L1 (SP263) assay (Roche Diagnostics) and the PD-L1-Tumor proportion score (TPS) was obtained. Patients were grouped into PD-L1 positive (PD-L1+) or negative (PD-L1-) profiles and stratified by sex and hormonal status. Patients with a PD-L1 TPS >1% were considered positive. A total of 95 tissues from NSCLC patients with a PD-L1+ profile and 72 from PD-L1- profiles were included.

### 2.3 Immunodetection of hormonal receptors

The expression of hormonal receptors ERα, ERβ, and AR was assessed by immunohistochemistry. Tissue serial sections (5μm) were obtained from the paraffin-embedded tissue blocks and placed on poly-L-lysine (Sigma-Aldrich) coated slides. Tissues were rehydrated and exposed to heat and pressure-induced epitope retrieval. Tumors sections were incubated with ERα (GTX22746 GeneTex), ERβ (ab187291 ABCAM) or AR (ab133273 ABCAM) antibodies at a concentration of 1:100, diluted in phosphate-buffered saline (PBS)-albumin, at 4°C overnight. An appropriate secondary antibody and HRP complex were used, and 3’3-Diaminobenzidine (DAB-Chromogen Kit System, Biocare Medical) was applied to visualize the reaction product. Tissues were washed in PBS after being treated with each reagent or antibody, and they were counterstained with hematoxylin and finally mounted. Prostate and uterine tissue sections were used as positive controls of hormonal receptors.

### 2.4 Immunostaining evaluation

In an independent and blinded analysis, two observers performed the immunoreactivity assessments of ERs and AR receptors in tumor samples from PD-L1 positive and negative patient groups. A tissue was considered positive, when an ochre color was observed in the nucleus or cytoplasm of tumor cells and negative for the absence of staining. The degree of positivity was determined by direct observation as weak staining (1+), intense staining (2+), and very intense staining (3+). A tissue was considered positive when it exhibited any grade of staining from weak to very intense.

In addition, tissue from each patient was analyzed and images of 5 random fields were captured using a digital camera (AXIOCAM208 COLOR) coupled to a light microscope (PRIMO STAR Zeiss) at 400x magnification. Fiji/ImageJ software was used to obtain an immunohistochemical staining score (IHC). Images were analyzed using the color deconvolution option; the threshold value was adjusted to eliminate the background signal without compromising the specific DAB signal. The mean grey value, representing the quantified signal (IHC), was obtained to investigate the protein expression levels in the PD-L1+ group (31, 32). Data from fields analyzed per patient was grouped and compared by sex and hormonal status (premenopausal women, postmenopausal women, and men) to identify whether significant differences existed.

### 2.5 Statistical analysis

We used R software (v4.2.3) (33) and specific R packages for all statistical analyses as indicated in each analysis. Statistical tests were selected based on the nature of the data and the study design, ensuring robustness and accuracy of the results. Parametric and non-parametric methods were applied according to data distribution, and advanced statistical models were used to evaluate associations, correlations, and effects adjusted for potential confounding factors. The chi-square test (χ²) for categorical data analysis was applied to assess whether PD-L1 status was significantly associated with the expression of each hormonal receptor. This test evaluates whether the observed frequencies of hormonal receptor expression differ significantly by PD-L1 profile. The dataset consisted of the proportion of patients expressing each receptor (ERα, ERβ, and AR) within the PD-L1- and PD-L1+ groups. To statistically evaluate these differences, we performed a Chi-square (χ²) goodness-of-fit test separately for PD-L1- and PD-L1+ groups to assess whether the distribution of receptor expression deviated significantly from an equal distribution. Given that some receptor subgroups contained low frequencies, Fisher’s exact test was also applied as a more robust alternative, particularly for small sample sizes, ensuring the accuracy of significance estimates (34, 35). Additionally, to determine whether ERβ expression was significantly higher than ERα and AR in PD-L1- patients, and whether ERα expression was considerably higher than ERβ and AR in PD-L1+ patients, odds Ratios (ORs) with 95% confidence intervals (CIs) were calculated. ORs provide a measure of how much more or less likely one receptor is expressed compared to another within a specific patient group (36)

Moreover, to quantify the strength of the association between PD-L1 status and each receptor, we implemented generalized linear models (GLMs) with a binomial logistic regression (37). This approach allowed estimating odds ratios (ORs), determining whether patients with specific receptor expressions were more likely to be PD-L1+. GLMs were particularly useful for adjusting for potential confounders (e.g., sex and hormonal status) and estimating the probability of being PD-L1+ based on receptor expression.

The co-expression patterns between ERα, ERβ, and AR were also investigated, analyzing whether the expression of one receptor correlated with the presence of another. This information was critical to determine whether hormonal receptors interact differently in PD-L1+ versus PD-L1- patients. A Chi-square test and Fisher’s exact test were performed to assess pairwise receptor associations, and the Phi correlation coefficient (φ) was calculated to quantify the strength of co-expression between two receptors (35, 38). The analysis was carried out using the “psych” package (39).

To investigate whether differences in ERα expression levels (IHC score) occur according to sex and hormonal status among PD-L1+ patients, a two-step statistical approach was performed. First, a global comparison among the three groups (premenopausal, postmenopausal, and male patients) was applied using the non-parametric Kruskal-Wallis test, which is robust in unbalanced designs with similar distribution shapes (40). When significant differences were detected, *post-hoc* pairwise comparisons were carried out using Dunn’s test with Bonferroni correction (41) using the R package “Dunn test” (42). To account for potential pseudoreplication, we complemented the non-parametric analysis by fitting a generalized linear mixed model (GLMM) in the R “lme4” package (43). This model accounted for intra-subject variability by including “Individual” as a random effect and was fitted using a Poisson distribution with a log link function (44). The significance of the fixed effect “Group” (i.e., hormonal status/sex) was evaluated via likelihood ratio tests (comparing the full model to a null model with only the random effect). In addition, bootstrap resampling (using 1,000 iterations) was applied to obtain 95% confidence intervals for the fixed effects, thereby providing an empirical validation of the statistical significance under the non-independence of measurements.

A correlation analysis explored the association between ERα expression and the PD-L1 TPS. PD-L1+ patients were stratified according to TPS, and ERα expression levels were compared across these strata. Finally, the correlation between ERα expression and clinical-pathological characteristics in PD-L1+ patients was investigated using Pearson’s correlation coefficient and linear mixed models to account for the unbalanced study design. Given the variability in the number of observations per patient, a linear mixed-effects regression model (LMM) was employed to assess the association between ERα expression levels (densitometry values) and smoking status, wood smoke exposure, asbestos exposure, and TNM classification, incorporating a random intercept for individual patients to mitigate intra-subject variability (43). Correlation matrices were visualized through heatmaps, while boxplots were utilized to compare ERα expression across risk factor categories. Regression coefficient plots were generated to illustrate the direction and magnitude of associations, including 95% confidence intervals. Statistical significance was defined as p < 0.05, and analysis was performed using the “lme4” package (43).

## 3 Results

**Figure 1:**
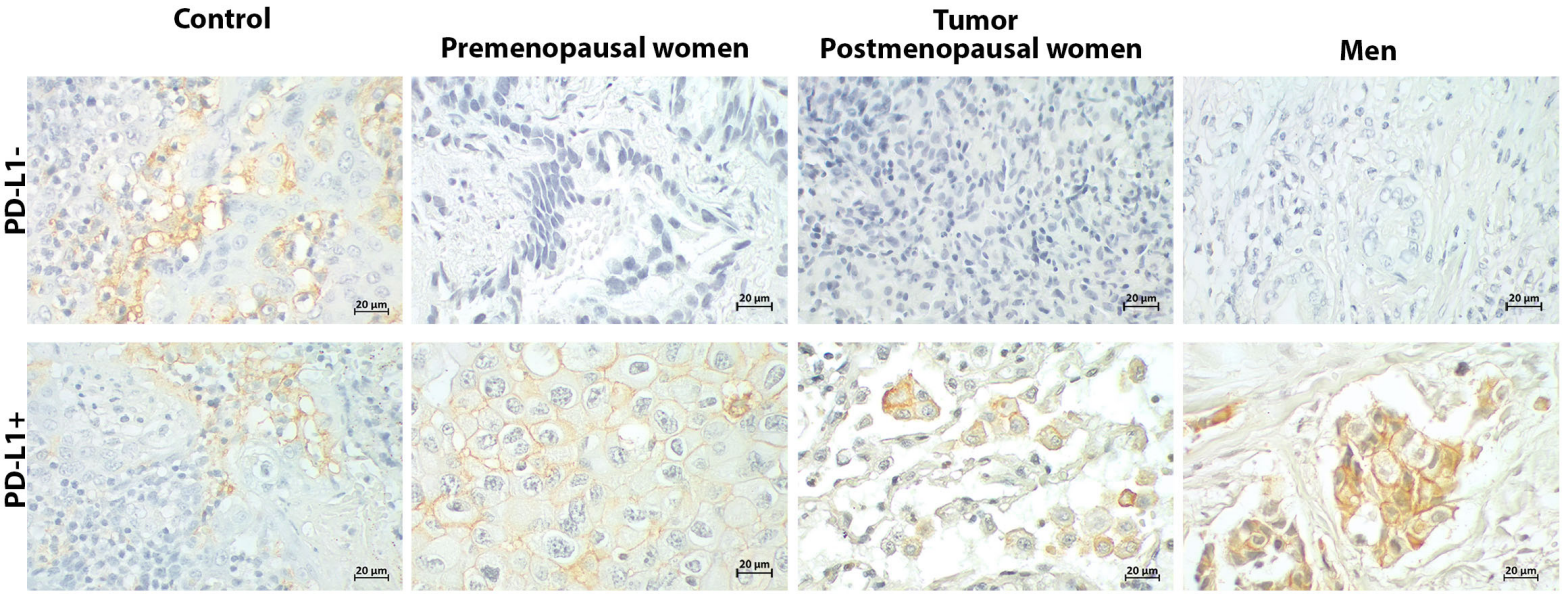
PD-L1 immunostaining of tumors from NSCLC patients. Tonsil was used as a positive control. The reactivity of PD-L1 was observed in the cell membrane of positive tissues.

The expression of hormonal receptors was analyzed by sex and hormonal status in NSCLC patients with PD-L1+ and PD-L1- profiles. A representative immunostaining of PD-L1 in patients who were considered negative and positive is shown in **Figure 1**. The PD-L1- group was used as a control to compare hormonal receptor expression in PD-L1+ patients. Clinical features of patients are summarized in **Tables 1** and **2**.

### 3.1 Expression patterns of hormonal receptors in tumors according to the PD-L1 profile

**Figure 2:**
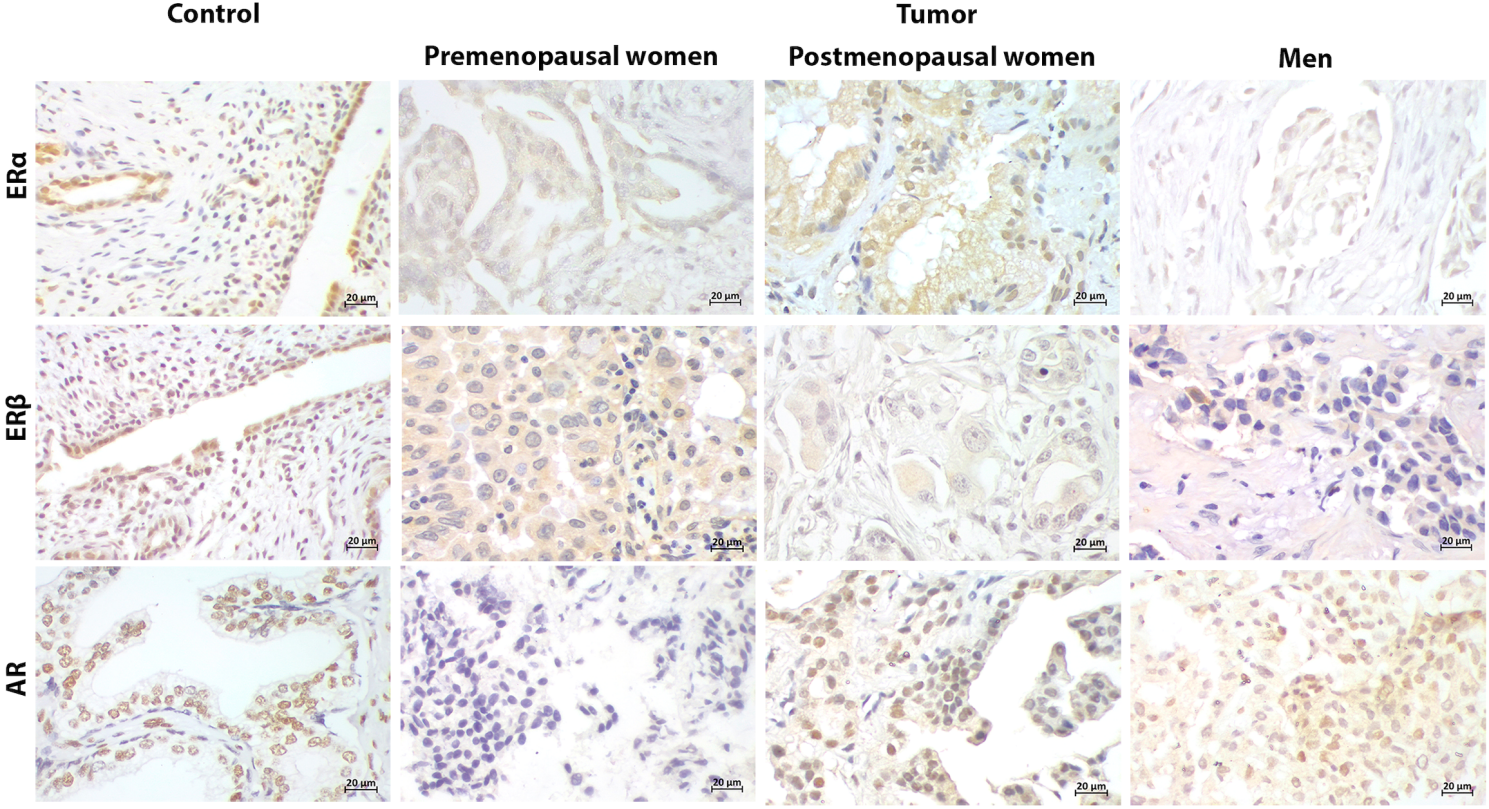
Representative immunostaining of hormonal receptors in PD-L1- patients. Uterus and prostate were used as positive control for estrogen receptors (ERα, ERβ) and androgen receptor (AR), respectively. Cellular localization of ERs and AR in control tissues and tumors was both nuclear and cytoplasmic. ERs were positive in tumors from patients regardless of the sex and hormonal status. AR was restricted to postmenopausal women and men (400x) (Scale bar 20μm).

**Figure 3:**
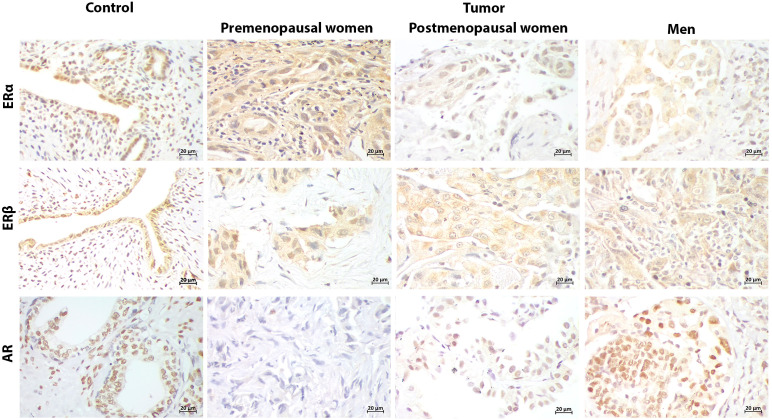
Representative immunostaining of hormonal receptors in PD-L1+ patients. Uterus and prostate were positive controls for estrogen receptors (ERα, ERβ), and androgen receptor (AR), respectively. Cellular localization of hormonal receptors in tumor was both nuclear and cytoplasmic, similarly to the PD-L1- group. ERs expression was widely distributed in tumors from patients regardless of the sex and hormonal status. AR expression was absent in premenopausal women regardless PD-L1 profile (400x) (Scale bar 20μm).

**Figure 4:**
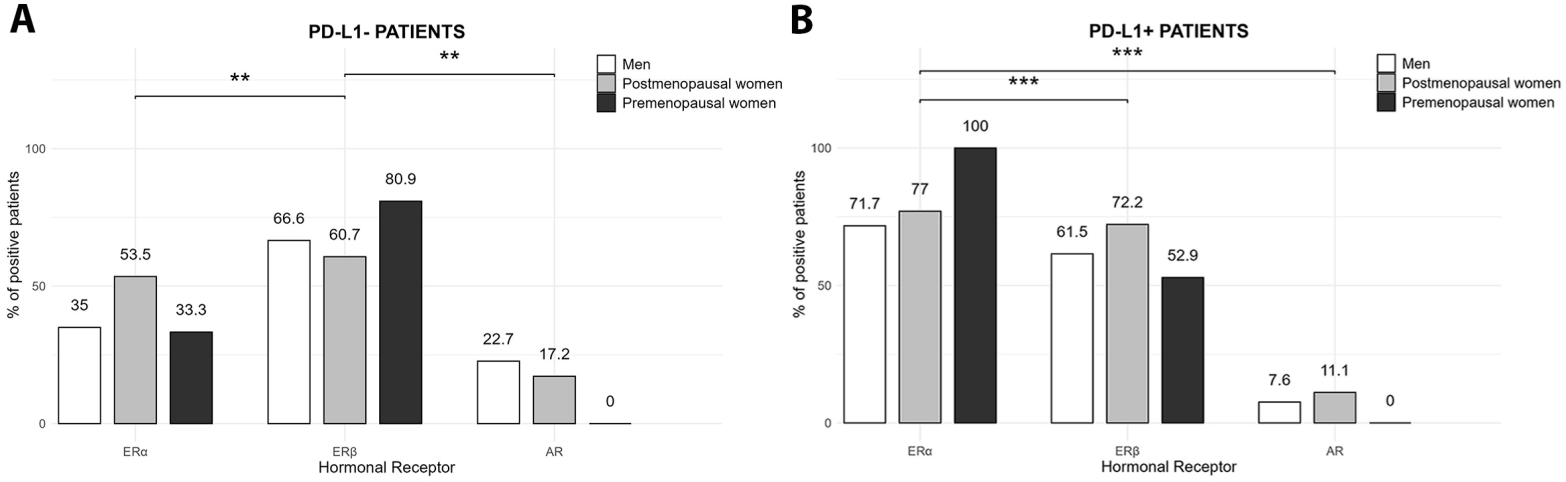
Expression of hormonal receptors in NSCLC patients according to the PD-L1 profile. **(A)** The PD-L1- group exhibited a significantly higher proportion of patients expressing ERβ compared to ERα and AR, both in women and men (**p<0.01, Chi-square (χ²) test). **(B)** The PD-L1+ group showed a significantly higher proportion of ERα-positive patients compared to ERβ and AR, regardless of the sex and hormonal status (***p < 0.001, Chi-square (χ²) test).

ERα, ERβ and AR expression was detected in both PD-L1 negative and positive patients (**Figures 2**-**4**). Immunoreactivity of hormonal receptors was detected in tumor cells’ nucleus and cytoplasm. The intracellular localization of these proteins was consistent among patients regardless of the sex, hormonal status, or PD-L1 profile (**Figures 2** and **3**).

However, patients’ hormonal receptor expression patterns differed according to the PD-L1 profile (**Figure 4**). In the PD-L1- group, ERβ was the predominant form of estrogen receptor expressed, with a prevalence of 80.9%, 60.7% and 66% in premenopausal and postmenopausal women and men, respectively. The proportion of patients with ERα expression was lower than ERβ, with 33.3% in premenopausal women, 53.5% in postmenopausal and 35% in men. The lowest expression among patients was observed for AR (0, 17 and 22% in premenopausal and postmenopausal women and men, respectively). In the PD-L1- group, the proportion of patients with positive signal for ERβ was significantly higher than ERα and AR regardless of sex and hormonal status (*p*<0.01) (**Figure 4A**).

On the other hand, in PD-L1+ patients, the main form of estrogen receptor expressed was ERα, occurring in 100% of premenopausal patients, 77% of postmenopausal females, and 71% of male patients. ERβ was expressed at a lower proportion than ERα, being positive in 52.9%, 72.2% and 61.5% of premenopausal and postmenopausal women, and men, respectively. AR showed the lowest expression, ranging from 0% of premenopausal females to 7.6% of postmenopausal females and 11% of male patients. The proportion of patients expressing ERα was significantly higher than those expressing ERβ and AR (*p*<0.001) (**Figure 4B**).

### 3.2 Hormonal receptors associated with PD-L1 profiles

Comparing the expression of hormonal receptors in patients according to their PD-L1 profile, we observed that the proportion of patients with ERα expression was higher in the PD-L1+ group compared to the PD-L1- group (*p*<0.0001). Moreover, a significant association was found between PD-L1+ status and ERα expression in both women and men (*p*<0.0001). The generalized linear model (GLM) further supported this finding, with an adjusted odds ratio (OR) of 5.01 for ERα, indicating that patients expressing ERα were five times more likely to be PD-L1+. The expression of ERβ and AR was similar among patients regardless of PD-L1 profile, and no significant associations were observed between PD-L1 status and ERβ (*p*=0.8194) or AR (*p*=0.399). AR expression in PD-L1- patients was primarily observed in males and was higher compared to the PD-L1+ group, indicating a potential sex-related regulation in this subset (**Table 3**).

**Table 3:** Expression and co-expression patterns of ERα, ERβ, and AR in NSCLC patients with PD-L1 positive and negative profiles.

Hormonal Receptor	PD-L1 profile	*p*	Men %	Women %	Women by hormonal status	
					PreMP %	PostMP %
ERα	–	***	35	44	33.3	53.5
	+		71.7	84	100	77
ERβ	–	–	66.6	69.3	80	60.7
	+		61.5	66	52.9	72.2
AR	–	*	22.7	10	0	17.2
	+		7.6	7.5	0	11.1
ERα/ERβ	–	***	15	30.6	28.5	32.1
	+		53.8	58.4	47	63.8
ERα/AR	–	–	5	0	0	0
	+		5	0	0	0
ERβ/AR	–	–	10	6	0	10.3
	+		0	0	0	0
ERα/ERβ/AR	–	–	5	1.5	0	3
	+		2.5	5	0	7

*P*-values correspond to overall comparisons of the total proportion of patients expressing each hormonal receptor between PD-L1- and PD-L1+ groups, regardless of the sex or hormonal status. Chi-square (χ²) tests was applied, and Fisher’s exact test was used for low frequencies. *Statistical significance: *p < 0.05, **p < 0.01, ***p < 0.001. The proportions of positive patients to hormonal receptors in PD-L1 groups, are presented by sex and hormonal status to show the different distribution in these subgroups, and to highlight the distinctive expression patterns in premenopausal women, a group of patients underrepresented in studies.

### 3.3 Co-expression of hormonal receptors in tumors according to PD-L1 profile

Additionally, the co-expression patterns of hormonal receptors were analyzed. Among PD-L1+ patients, the co-expression of ERα and ERβ was significantly higher than among PD-L1- patients (*p* < 0.0001). A positive association was observed between ERα and ERβ (*p* = 0.00085, odds ratio = 6.35, φ coefficient = 0.38), while AR did not exhibit co-expression with ERα in female patients and was detected in only 5% of male patients, regardless of PD-L1 status. AR/ERβ co-expression was observed exclusively in postmenopausal women and men in the PD-L1- group. Finally, ERα/β and AR co-expression was low and restricted to male and postmenopausal female patients (**Table 3**).

### 3.4 Expression levels of ERα in PD-L1 + patients

**Figure 5:**
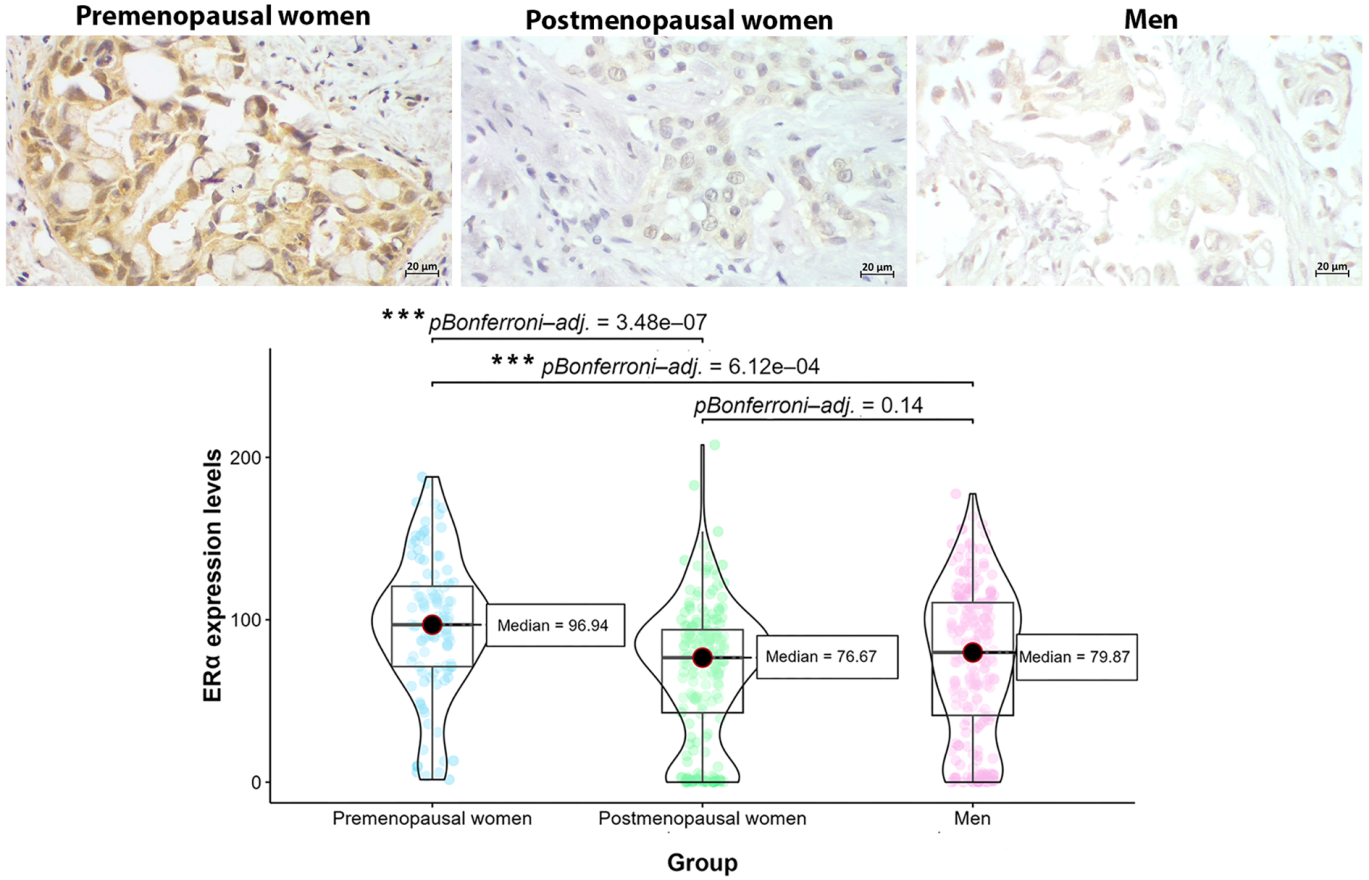
Level expression of ERα in tumors from PD-L1+ patients by sex and hormonal status. Premenopausal women exhibited the highest levels of ERα expression compared to postmenopausal women and men, suggesting the effect of hormonal status in ERα upregulation (***p<0.0001, Kruskal-Wallis test and *post-hoc* Dunn’s test with Bonferroni correction).

Since ERα was the predominant form of hormonal receptor in PD-L1+ patients and was strongly associated with PD-L1+ profile, we analyzed the expression levels of this receptor by densitometry, comparing them across sex and hormonal status. The densitometric analysis revealed that, among PD-L1+ patients, the highest levels of ERα expression were observed in premenopausal women and male patients (**Figure 5**). However, the global comparison using the Kruskal-Wallis test showed statistically significant differences in ERα expression levels between groups (Kruskal-Wallis χ² = 28.24, df = 2, *p* < 0.0001), with *post-hoc* Dunn’s tests indicating that the difference was significant only for premenopausal women compared to the other groups. Complementary analysis with the GLMM further supported these findings. The model, which included “Group” as a fixed effect and “Individual” as a random effect, yielded a significant effect of group on ERα expression (likelihood ratio test: χ² = 3.35, df = 2, *p* = 0.01874). Bootstrap resampling of the fixed effects provided 95% confidence intervals that did not include zero for the premenopausal group (CI: 0.399, 0.490), confirming a significantly higher expression level in this group compared to the other groups.

### 3.5 Relationship between ERα expression, PD-L1 TPS and clinical features

The association between ERα expression and PD-L1 TPS was also investigate; however, the analysis demonstrated no significant correlation (*p* < 0.05), suggesting that the expression of ERα is associated with PD-L1 + profile but is independent of the PD-L1 TPS score.

Additionally, the linear mixed-effects model analysis, accounting for intra-patient variability, did not reveal any statistically significant associations between ERα expression levels and the clinical-pathological factors evaluated (smoking status, wood smoke exposure, asbestos exposure, and TNM classification). The model’s AIC (2759.4) and BIC (2785.2) indicate a moderate fit, and the random effects analysis suggested that inter-patient variability (variance = 1577.4, SD = 39.72) plays a significant role in ERα expression levels. Among the fixed effects, smoking showed a weak negative association with ERα expression (β = -7.55, SE = 11.01, t = -0.685), while TNM staging exhibited a weak positive association (β = 6.59, SE = 10.10, t = 0.653). However, none of these relationships were statistically significant. Similarly, wood smoke exposure (β = 1.25, SE = 11.76, *t* = 0.106) and asbestos exposure (β = -0.54, SE = 25.61, *t* = -0.021) did not show significantly correlate with ERα expression. Correlation analysis revealed weak but non-significant correlations (*p* < 0.05) among some variables. Specifically, ERα level expression exhibited weak negative correlations with smoking (*r* = -0.097) and weak positive correlations with TNM staging (*r* = 0.101) and asbestos exposure (*r* = 0.093). Nevertheless, these small effect sizes suggest that the observed relationships are unlikely to be clinically meaningful.

## 4 Discussion

NSCLC exhibits distinct biological and clinical behaviors between women and men, although sex bias in responses to immunotherapy needs further investigation; several studies have shown that sex hormones, through their receptors, play a pivotal role in lung carcinogenesis, the clinical progression of the disease, patient survival, and treatment response (25, 45–48). Estrogen has been shown to exert a significant influence on lung cancer due to the widespread expression of its receptors and its capacity to promote carcinogenic processes, including immune modulation. Several studies have reported that the predominant form of ER expressed in lung cancer is ERβ, which is overexpressed in 50-80% of tumors, while positivity for ERα occurs in 30-50% of tumors (49–54). Our results support these observations, since the main hormonal receptors expressed were estrogenic, both in women and men, regardless of PD-L1 profile. We also observed that ERβ was the predominant form of ER in PD-L1- patients (control group), exhibiting in 60-80% of patients while ERα was the less common form, found in 33.3-53.5% of patients, as described in the literature (49, 52).

However, we describe for the first time a different pattern of hormonal receptor expression in PD-L1+ patients who exhibited ERα as the predominant form of estrogen receptor. When compared with the PD-L1- group, positivity for ERα significantly increased in PD-L1+ patients. Additionally, a significant association between ERα+ and PD-L1+ was found, suggesting a relationship between these proteins.

Estrogens orchestrate lung cancer progression differently depending on the type of estrogen receptor expressed. ERβ signaling has been associated with enhanced cell proliferation, cell survival, tumor growth, angiogenesis, migration and metastasis (25). In contrast, ERα signaling is linked to increased tumor invasion via MMP9 production, activation of the CXCL12/CXCR4 pathway and the promotion of M2 macrophage polarization and infiltration (55). While the biological role of ERβ in NSCLC has been relatively well established, the significance of ERα remains under investigation. Nevertheless, our findings suggest that ERα may play a distinct role in modulating the tumor microenvironment, potentially contributing to immune escape mechanisms through PD-1/PD-L1 axis.

PD-L1 expression in NSCLC is influenced by a complex network of intrinsic and extrinsic regulatory mechanisms, many of which interact with key oncogenic and inflammatory pathways. In the context of our findings, the possibility that ERα modulates PD-L1 expression aligns with current knowledge about its dynamic regulation. Intrinsically, PD-L1 is transcriptionally controlled by oncogenic drivers such as MYC, KRAS, EGFR, and ALK, as well as epigenetic regulators like DNA methyltransferase-1 (DNMT1), histone deacetylases (HDACs), and microRNAs including miR-3127-5p and miR-142. Post-translational modifications, such as phosphorylation, glycosylation, ubiquitination, and palmitoylation, also contribute to its stabilization and membrane localization. In addition, several extrinsic factors including cytokines (e.g., IFN-γ, TNF-α, IL-1α), growth factors (EGF, TGF-β, VEGF), hypoxic stress via HIF-1α, and exposure to radiation or chemotherapy have been shown to modulate PD-L1 levels (56, 57). These multilayered regulatory mechanisms support the plausibility of hormone-related modulation and highlight the need to further investigate ERα’s involvement in shaping the immune landscape of NSCLC.

In hormone dependent cancers such as breast and endometrial, the relationship between PD-L1 and estrogen has been analyzed, but results are still controversial. Some studies showed that E2 negatively regulated PD-L1 and antiestrogen increase PD-L1 expression (58, 59), while other reported the up-regulation of PD-L1 by E2/ERα signaling (60, 61). These discrepancies exhibit the complex and context-dependent nature of PD-L1 regulation, which may vary depending on cancer type, mutational landscape, and tumor microenvironment. Importantly, several components of the tumor microenvironment, particularly those involved in immune modulation, are known to be influenced by estrogen signaling (62).

Among these, INF-γ, a potent inductor of PD-L1 is regulated by estrogen pathway as was observed in thymocytes and natural killer cells in the spleen. E2 through both ERα and ERβ accelerated the transcriptional activity of IFN- γ mRNA (58, 59). Furthermore, it has been reported that E2 directly affects IFN-γ promoter activity (63).These findings support a mechanistic link between hormonal signaling and immune checkpoint regulation, which may be particularly relevant in NSCLC tumors expressing high levels of ERα.

Likewise, crosstalk between E2/ER and EGF/EGFR pathways has been previously described in NSCLC. Treatment with E2 increased EGF production, and ER transactivated the EGFR in a non-genomic mechanism. In turn, EGFR activation enhances aromatase expression and activity, further amplifying estrogen production (26, 64, 65). Moreover, VEGF expression is a downstream result of EGFR activation induced by estrogen (66). Both EGFR and VEGF pathways have been implicated in regulation of PD-L1 expression. These findings suggest that E2 may influence PD-L1 expression indirectly through modulation of EGFR and VEGF pathways in NSCLC.

In addition to growth factor signaling, hypoxia upregulates PD-L1 expression in cancer cells through hypoxia inducible factor HIF-1α (67). In breast cancer cells, it was reported that HIF-1α gene expression was under the control of estrogen directly through ERα, since this gene contains an estrogen response element (68). Similarly, in NSCLC a strong association between ERβ and HIF-1α has been identified (69), indicating a potential mechanism by which E2 may influence PD-L1 expression under hypoxic conditions. These interactions further reinforce the hypothesis that hormonal signaling, particularly via ERα and ERβ, contributes to the immune landscape of NSCLC by modulating PD-L1 expression through multiple, context-dependent molecular pathways.

Taken together, these findings suggest that estrogen-associated pathways may converge to influence PD-L1 expression in NSCLC. Despite the growing body of mechanistic evidence, the direct relationship between sex hormones and the PD-1/PD-L1 immune axis in lung cancer remains poorly understood, even though sex-based differences in immunotherapy outcomes have been consistently observed in clinical settings.

More recently, two studies have provided novel evidence supporting a direct regulatory link between estrogen signaling and PD-L1 in lung cancer. In NSCLC cell lines, estradiol was shown to increase sirtuin 1 (SIRT1) expression via ERβ, leading to elevated PD-L1 levels (70). This axis may represent a previously unrecognized mechanism of immune evasion driven by estrogen, further underscoring the potential impact of hormonal signaling on the tumor immune environment in NSCLC.

Moreover, in 2023 Anobile and coworkers (71) found that ERα was a predictive factor of response to anti-PD-L1 inhibitors, more potent than sex and PD-L1 levels; this effect was predominant in female patients. In NSCLC cell lines, E2/ERα pathway upregulated PD-L1 through EGFR/Akt and ERK1/2. Additionally, treatment with fulvestrant and letrozole (estrogen receptor and aromatase inhibitors respectively), reduced PD-L1 expression. In this study ERβ was transcriptionally less active than ERα (71). Also, the relationship between ERα and PD-L1 was described in breast cancer. Estradiol upregulated PD-L1 but not PD-L2, through the PI3K/Akt pathway only in MCF-7 and Ishikawa ERα positive cells (60). Our results are consistent with these findings, as we observed a stronger relationship between ERα and PD-L1 positivity regardless of sex and hormonal status in NSCLC patients. In contrast, ERβ and AR showed no significant relationship with PD-L1 status, underscoring the central role of ERα in PD-L1 regulation.

In addition to individual receptors expression, we examined the co-expression patterns of ERα and ERβ in relation to PD-L1 status, a topic that has not been previously addressed in NSCLC. The co-expression of estrogen receptors was low in the PD-L1- group but increased in the PD-L1+ group, supporting a significant role of estrogen pathway in PD-L1 control. This co-expression may reflect a more aggressive, immunoevasive tumor phenotype, as each receptor subtype activates distinct biological processes. In breast cancer, ERα/ERβ co-expression has been linked to unfavorable features such as higher histological grade, lymph node involvement, HER2 negativity, and increased Ki-67 expression (72). However further studies are needed to elucidate the biological and clinical significance of estrogen receptor co-expression in lung cancer and its relation to PD-L1 axis and immune escape mechanisms.

On the other hand, androgens signaling via AR has also been implicated in lung carcinogenesis, although AR expression appears to be relatively uncommon in NSCLC. Our results showed that AR was presented in 0-22.7% of patients in the PD-L1- group, which is consistent with the literature where AR has been identified in 20% of LC patients (73). Additionally, we reported that the prevalence of AR in the PD-L1+ group was lower than in the PD-L1 negative group, ranging from 0-11% of patients. These findings suggest that AR may play a limited, context-specific role in NSCLC, possibly restricted to a distinct molecular or clinical subset of patients.

Although the role of AR in lung cancer remains controversial, some studies have shown that it promotes cell proliferation, migration and invasion. Also, the KRAS mutational profile has been linked to AR levels in NSCLC. Nevertheless, its function in immune evasion in LC has not been explored, our results suggest that AR does not have a relevant function in PD-L1 control in NSCLC.

Despite evidence that male patients tend to respond better to PD-L1-based immunotherapy (15, 18, 20, 74), androgens through AR do not appear to be involved in PD-L1 expression. Our results showed that a low percentage of patients in PD-L1+ group expressed AR, and no association was found between AR and PD-L1+ profile in NSCLC.

Studies in other cancer types have shown a negative association between AR and PD-L1 expression. For instance, a negative correlation between AR and PD-L1 expression was reported in tumors from patients with hepatocellular carcinoma, and increased lymphocyte infiltration was observed in AR- negative tumors treated with anti-PD-L1 drugs compared to AR-positive tumors (75). Similarly, AR was negatively correlated with PD-L1 in thyroid cancer, where AR reduced PD-L1 promoter activation by NF-kβ signaling (76). Moreover, AR regulated the immune response to tumors in bladder cancer by downregulating PD-L1 by directly binding to AR response elements of the PD-L1 promoted region and increasing CD8+ lymphocyte activity (77). Our results are consistent with these findings in other cancer types, as no association was found between AR expression and the PD-L1+ profile in NSCLC patients, suggesting that AR does not promote immune escape through up-regulation of PD-L1. However, additional studies are necessary to clarify the role of AR in lung carcinogenesis including its involvement in immune escape mechanisms.

In LC, the estrogen pathway, mainly through ERα, appears to play a more critical role in PD-L1 expression than androgens in women and men. Nevertheless, differences by sex in PD-L1 expression and response to immunotherapy, might be explained by multiple factors other than sex hormones, such as factors inherent and related to sex. Smoking habits, mainly associated with men, have been related to PD-L1 expression (78, 79). Besides, KRAS mutation, frequently associated with smokers, has also been linked to PD-L1 expression in lung cancer (80).

Finally, our results revealed that ERα expression was significantly higher in PD-L1+ premenopausal women, compared to postmenopausal and men. This pattern may be attributable to higher circulating estradiol levels in premenopausal women, in combination with local intra-tumoral estrogen production, which could stimulate ERα expression, as ER is an estrogen-inducible gene (81, 82). These findings highlight a potential therapeutic implication; PD-L1+ patients, particularly premenopausal women, may benefit from combination strategies that integrate immune checkpoint blockade with anti-estrogen therapies. Future studies are needed to explore this approach and evaluate its efficacy in a clinical setting.

## 5 Conclusions

In summary, our findings demonstrate that estrogens primarily through ERα, play a significant role in regulating PD-L1 expression in LC, affecting both male and female patients. Although men respond better to PD-L1 inhibitors, the androgen pathway was not associated with PD-L1 expression.

Estrogen signaling pathway, by contrast represents a promising therapeutic target to enhance the effectiveness of PD-L1 inhibitors, regardless of sex and hormonal status. Estrogen receptor expression could be considered among tumor biomarkers for optimizing immunotherapy strategies in both women and men.

Additionally, premenopausal women are frequently underrepresented in studies, but the hormonal effects in these patients are stronger compared to postmenopausal women, and men, as indicated by higher estrogen receptor expression in tumors from PD-L1+ patients, likely driven by elevated circulating estrogen levels. Although antiestrogen treatment could improve immunotherapy both in women and men, premenopausal women might benefit significantly from combined antiestrogen and PD-L1 inhibitors. Further studies are needed to fully understand the immune evasion mechanisms mediated by the estrogen pathway, including the regulation of PD-L1, and to clarify their potential impact on immunotherapy response.

## Data Availability

The original contributions presented in the study are included in the article/supplementary material. Further inquiries can be directed to the corresponding author/s.
